# Relevance of Topographic Parameters on the Adhesion and Proliferation of Human Gingival Fibroblasts and Oral Bacterial Strains

**DOI:** 10.1155/2019/8456342

**Published:** 2019-02-10

**Authors:** Miguel Ángel Pacha-Olivenza, Ricardo Tejero, María Coronada Fernández-Calderón, Eduardo Anitua, María Troya, M. Luisa González-Martín

**Affiliations:** ^1^Department of Biomedical Sciences, Faculty of Medicine, University of Extremadura, Avda de Elvas s/n, 06006 Badajoz, Spain; ^2^Networking Research Center on Bioengineering, Biomaterials and Nanomedicine (CIBER-BBN), Avda de Elvas s/n, 06006 Badajoz, Spain; ^3^BTI Biotechnology Institute IMASD, 01510 Miñano, Spain; ^4^Private Practice in Implantology and Oral Rehabilitation, 01007 Vitoria-Gasteiz, Spain; ^5^Department of Applied Physics, Faculty of Science, University of Extremadura, Avda de Elvas s/n, 06006 Badajoz, Spain

## Abstract

Dental implantology allows replacement of failing teeth providing the patient with a general improvement of health. Unfortunately not all reconstructions succeed, as a consequence of the development of infections of bacterial origin on the implant surface. Surface topography is known to modulate a differential response to bacterial and mammalian cells but topographical measurements are often limited to vertical parameters. In this work we have extended the topographical measurements also to lateral and hybrid parameters of the five most representative implant and prosthetic component surfaces and correlated the results with bacterial and mammalian cell adhesion and proliferation outcomes. Primary human oral gingival fibroblast (gum cells) and the bacterial strains:* Streptococcus mutans*,* Streptococcus sanguinis* and* Aggregatibacter actinomycetemcomitans*, implicated in infectious processes in the oral/implant environment were employed in the presence or absence of human saliva. The results confirm that even though not all the measured surface is available for bacteria to adhere, the overall race for the surface between cells and bacteria is more favourable to the smoother surfaces (nitrided, as machined or lightly acid etched) than to the rougher ones (strong acid etched or sandblasted/acid etched).

## 1. Introduction

Dental implantology allows immediate replacement of failing teeth. Besides recovering aesthetics and masticatory functions, oral reconstructions are often associated with general health improvements [1], but unfortunately not all reconstructions succeed. Statistics on dental implants show that success rate declines over time, especially in the presence of specific systemic conditions [2, 3]. Very often, the failures are linked to the development of infections of bacterial origin at the implant surfaces that run out of the control of clinicians. To cause infection, bacteria must first colonise and then be retained at the implant site. Therefore, the challenge persists in the design of implants with not only sufficient mechanical and integrative capacities, but also resistant to bacterial infections.

Implant surface topography has historically been in the spotlight since it is known to modulate a differential response to bacterial and mammalian or eukaryotic cells. Differences in topography are sensitive to the different sizes and external membrane compositions of these cells. More especifically, bacteria membranes are rigid and can be formed either by a thick external peptidoglycan layer, which defines a Gram-positive bacteria type or by a thin peptidoglycan layer covered by polysaccharides, which defines a Gram-negative type. In both cases, and specifically for colonizers on both hard and soft tissues in the oral cavity, the total diameter is around 1 *μ*m [4, 5]. Bacterial membrane rigidity may hinder their interaction with complex topographies, especially on nano-sized topographies on which the bacterial size exceeds the size of the accessible adhesion cavities. On the other hand, eukaryotic cells, like gingival fibroblasts possess a very elastic and flexible external membrane prone to accommodate to complex surface topographies and allow up to 100 *μ*m cytoplasm spreading [6].

Topography can be characterized by a set of parameters that give information about height and distribution of the features on the surface. The arithmetical mean roughness, Sa (Ra in profile), and the root mean square roughness, Sq (Rq in profile), are the most used amplitude parameters [7]. However, these provide only a description of vertical measurements, height and depth of the peaks and valleys, but lack information about their distribution. Therefore a proper topographical description should also include lateral and hybrid parameters [8]. Also, the topography of a surface varies enormously depending on the scale chosen by the observer. Three categories of surface roughness, termed as macro (Ra ~ 10 *μ*m), micro (Ra ~ 1 *μ*m) and nanoroughness (Ra ~ 0,2 *μ*m), have been proposed. Atomic force microscopy and optical and stylus profilometers are commonly used to characterize topography. Nevertheless, Sa and Sq values of a given surface obtained with these techniques can yield different results due to the typical dimension of the actual element used by the instrument to measure, i.e., the radius of a tip or the wavelength chosen, and the size of the scanned area [9, 10].

Multiple studies have characterized the relationship between topography and adhesion of mammalian cells and bacteria. Roughened surfaces influence microbial colonisation and osseointegration in different ways. On the one hand, microbial retention is improved with surface irregularities [11] while on the other osteogenic differentiation is improved and stress distribution is optimized [12, 13]. Regarding bacterial adhesion, some authors found adhesion to increase with Sa and relate it to nanoroughness, whereas others reported a decrease in cases with a different range. It is generally accepted that a threshold value of 200 nm represents the average roughness (Ra) below which bacterial adhesion cannot be further reduced [14, 15]. Carlen et al. [16] found that Ra increments of 150 to 560 nm increased the numbers of adhering* Streptococcus sanguinis* and* Streptococcus mutans* on the surfaces of composite resin. Kawai et al. [17] also observed similar trends for Ra increments of 40 to 1240 nm in the adhesion of* Pseudomonas aeruginosa and Staphylococcus epidermidis* on acrylic surfaces and Boyd et al. [18] found that enhanced adhesion of* Staphylococcus aureus* occurred on rougher stainless steel compared to its adhesion on smooth surfaces. However, S. Shaikh et al. [19] found that Ra increments of 1880 to 6250 nm significantly reduced the adhesion of* S. aureus*,* P. aeruginosa*, and* Escherichia coli* on the surfaces of bioactive glases. Similarly, Taylor et al. [20] reported increase in* P. aeruginosa* adhesion with Ra increments of 40 to 1240 nm, although in the same report it was observed that bacterial adhesion decreased notably when Ra was increased of 1860 to 7890 nm. Surfaces with features on the same scale as bacteria cells appeared to promote the strongest attachment due to maximal cell-substrate contact area [21]. Regarding mammalian cells, Grössner-Screiber et al. found more adhesive contacts of fibroblasts on titanium smooth surfaces being the roughness characterized by Ra and measured with a mechanical stylus [22]. On rougher surfaces, cell spreading requires actin microspikes at the leading edges of lamellipodia to bend in energetically unfavourable ways that inhibit spreading [23]. In addition, Pierres et al. proved that a series of surface testing mechanisms precede cell adhesion. Fast and small fluctuations of the external membrane sense the presence of surfaces at a distance of at least 50 nm and monitor the topographical environment before adhesion occurs [24]. This process begins with the contact of the microvillis tip to a limited area of the surface and continues with the cell flattening in an area close to that of the cell size. [25]. Therefore, the height and spatial distribution of the topographical features play an important role in the adhesion performance.

The oral cavity is one the most complex and populated microbial niche in the human body. Several hundred different microorganisms are present in this environment and their different specialization allows them to live in either aerobic or anaerobic conditions inside the biofilm that constitutes the dental plaque. Some of these species are especially relevant because of their roles in the oral infective processes. In this work, we have considered* Streptococcus mutans*,* Streptococcus sanguinis*, and* Aggregatibacter actinoycetemcomitans* as relevant and representative of this environment.* S. mutan*s is considered to be the primary etiological agent of human dental caries [26, 27] and is part of the 20% of the Streptococci present in oral biofilm [28].* S. sanguinis* is one of the earliest microorganisms involved in the formation of the dental plaque and serves as attachment for other colonists.* A. actinoycetemcomitans* is found among the last species arriving to the dental plaque and, although it is also part of the normal human oral microflora, it is also strongly related to periodontitis with a high adhesive capacity [29].

The aim of this work is to analyse the behaviour of primary human oral gingival fibroblast and three bacterial strains implicated in the oral biofilms on five different and representative implant surfaces. We seek to ascertain how fibroblast adhesion and proliferation, and bacterial adhesion and biofilm formation are related to their surface topographical parameters.

## 2. Materials and Methods

### 2.1. Surface Preparation and Characterization

One 3 m-long bar of commercially pure titanium grade IV was used to produce a total of 525 discs of 12.7 mm diameter and 2 mm height. The as machined discs (Mach) were degreased and cleaned prior subsequent use. Physical Vapor Deposition (PVD) technique was used to produce nitrided samples (TiN) on Mach substrates using a titanium target in a nitrogen-rich atmosphere. Plasma sublimation permits the positive ionisation and an electric field imposed in the substrate allows the deposition of a 2-3 *μ*m thick homogeneous layer of titanium nitride. The process is maintained for 6h at 480°C to assure an optimum adhesion of the coating. Sandblasted and acid etched (SB+AE) surfaces were first bombarded with large Al_2_O_3_ particles (250 *μ*m average diameter) at 20 mm from the surfaces, during 20 s and at 7 bar pressure and secondly, and immersed in a mixed solution of concentrated H_2_SO_4_/HCl at 90°C for 5 min, followed by HNO_3_ 15% passivation for 20 min. Discs were then cleaned and conditioned in a clean room class A before sterilization under ß radiation. AEn and AEt surfaces were prepared similarly but without sandblasting and exposed to the acid bath for 10 min and 40 min, respectively (BTI Biotechnology Institute S.L., Vitoria, Spain).

#### 2.1.1. Surface Chemical Composition

X-ray photoelectron spectroscopy (XPS) measurements were performed using a K-Alpha (Thermo Scientific instrument, Waltham, MA, USA) equipped with a monochromatic AlK*α* X-ray source (1486.7 eV). The samples were investigated under ultrahigh vacuum conditions (3.5 10^−8^ mbar). The X-ray spot size was 300 *μ*m. Survey spectra of the samples were collected and used to calculate atomic percentages of the elements present on the surface. The pass energy for the survey spectra was 200 eV. The binding energy (BE) values were referenced by setting the C1s BE to 285.0 eV and the XPS spectra were background subtracted using Shirley method [30].

#### 2.1.2. Surface Roughness Evaluation

The roughness of the surfaces was measured by optical profilometry (3D Sensofar Pl*μ*, Terrasa, Spain). A Gaussian filter that consists in a continuous convolution that use the Gaussian 3D shape as weight function is employed, which is the most relevant to characterize highly periodical morphological structures [31]. This technique is standardized (ISO 25178), and the cut-off has been taken as 20 x 20 *μ*m on the primary surface to split the information between short-wave roughness and long-wave waviness. The areas scanned were 249 x 187 *μ*m. Results are averaged from 6 measurements per surface condition.

#### 2.1.3. Saliva Conditioning

To condition surfaces with human saliva, whole and unstimulated natural saliva samples collected in sterile plastic tubes were obtained from healthy volunteers at least 1.5 h after eating, drinking, or tooth brushing. Protocol of saliva preparation for experiments was done in accordance with Sánchez et al [32]. Then aliquots of 10.0 mL were treated as described [33]. Previous to bacterial tests, a set of samples of each of the different surfaces analyzed was conditioned in natural saliva. To this purpose, samples were covered with 400 *μ*l of natural saliva and left in contact for 60 min, under sterile conditions.

Artificial saliva was prepared according to the procedure described by Gal et al [34]. It is a solution at pH 6.8 composed of NaCl (125.6 mg L^−1^), KCl (963.9 mg L^−1^), KSCN (189.2 mg L^−1^), KH_2_PO_4_ (654.5 mg L^−1^), Urea (200.0 mg L^−1^), Na_2_SO_4_ 10 H_2_O (763.2 mg L^−1^), NH_4_Cl (178.0 mg L^−1^), CaCl_2_2H_2_O (227.8 mg L^−1^), and NaHCO_3_ (630.8 mg L^−1^).

### 2.2. Cell Studies

Primary human gingival fibroblasts were isolated as previously described [35] and used to assess the influence of different surfaces on cellular behaviour.

Gingival fibroblasts were maintained as stated previously [33] but here cells between the fourth and the fifth passage were used in the experiments.

#### 2.2.1. Adhesion and Proliferation

The adhesion and proliferation experiments were carried out as described in [33] but here we investigated also 30, 60, and 90 min of adhesion.

#### 2.2.2. Extracellular Matrix Protein Release

The discs with the different surfaces were placed on tissue-culture polystyrene plates. Cells were seeded in complete medium at a density of 6000 cells·cm^−2^. Cells cultured on tissue-culture polystyrene were used as control samples. After 7 days of culture, ELISA kits (Takara, Shiga, Japan) were used to determine both the fibronectin and the procollagen type I synthesis on fibroblast-conditioned medium.

### 2.3. Microbial Studies

The oral strains used for adhesion and biofilm formation assays were* S. mutans* ATCC 25175,* S. sanguinis* ATCC 10556, and* A. actinomycetemcomitans* ATCC 43718. From the frozen stock, bacteria were inoculated and incubated in Brain-Heart Infusion agar or broth (BHI, PanreacQuímica S.A., Spain) at 37°C in 5% CO_2_ (Galaxy® 170S, Eppendorf AG, Hamburg, Deutschland) for* Streptococci* strains and in anaerobic conditions for the* Aggregatibacter* strain (Whitley A35 Anaerobic Workstation, Don Whitley Scientific Limited, West Yorkshire, UK). 50 mL BHI was inoculated during 24 h in the case of the* Streptococci* strains and 100 mL BHI during 18 h in the case of the* Aggregatibacter* strain.

#### 2.3.1. Adhesion

Bacterial adhesion experiments were carried out following the experimental procedures previously described [33]. In short, we used a modified robbins device to keep in contact for 60 min the titanium samples with a bacterial suspension (3 x 10^8^ bacteria mL^−1^) collected in their exponential phase of growth, under laminar flow conditions (2 mL min^−1^) Then, the bacterial flow was carefully stopped, the samples removed, and the retained bacteria quantified. The adhered microorganisms were stained with Live/Dead Baclight L-7012 kit (Invitrogen SA, Spain) and counted automatically with the software NIS-Elements BR 4.10 (Nikon Instruments INC., USA) under epifluorescence microscopy. All the experiments were repeated three times with independent bacterial cultures.

#### 2.3.2. Biofilm Formation

For the biofilm formation assays, the previously described protocol was followed [33]. In short, biofilm production of bacteria adhered to the surfaces was allowed for 24 h at 37°C using BHI as culture media. After this period, biofilms were removed and the viable bacteria within the biofilm were quantified with the BacTiter-Glo™ Microbial Cell Viability Assay (Promega Corporation, Madison, WI, USA) according to the manufacturer's instructions. Samples were transferred to new culture plates, and the BacTiter-Glo™ reagent was added and allowed to act in the dark for 15 min at 20 RPM. The supernatant was transferred to white polystyrene flat-bottomed microtiter plates (Greiner bio-one) and the light emission reaction (luciferin-luciferase) was measured by a luminometer (Microplate Fluorescent Reader FLX 800, Bio-Tek Instruments, USA). Each assay was performed in duplicate and repeated three or more times with independent cultures in order to confirm reproducibility.

#### 2.3.3. SEM Images

Scanning electron microscopy (SEM) was used to obtain micrographs of the biofilm morphology on the surfaces. Samples were fixed in 3% glutaraldehyde for 12-15 h at room temperature, washed with PBS (pH 7.4) and dehydrated through a series of graded ethanol solutions (30%, 50%, 70%, 90%, and 100%) for 1 h each, and air-dried. The samples were subsequently vacuum-dried, sputter-coated with Au, and observed using a scanning electron microscope Quanta 3D FEG (FEI, Hillsboro, US). To acquire secondary electron images, low vacuum conditions and 15 kV of voltage were used. The samples were surveyed at magnifications ranging from 150 to 50,000 at random locations on the different samples.

### 2.4. Statistics

The data is expressed as means ± standard deviation of at least three independent experiments. The distribution date for each sample was Gaussian and the differences between the means were determined by one-way analysis of variance (ANOVA) using a software package (SPSS for Windows, release 19.0; SPSS, Chicago, IL). Statistical significance was accepted for p<0.05 after comparing the mean values by the Bonferroni and Tukey HSD test. Also, T-test was employed in the analysis of the differences between the means of two independent populations (“Uncoated” and “Human saliva coated”) during biofilm formation.

## 3. Results

### 3.1. Surface Characterization

XPS results are summarized in Table 1. Titanium dioxide is the main constituent in all surfaces. Putative carbon from environmental contamination is also present as well as some minute amounts of other elements. N at TiN (28%) is due to the PVD coating. A small amount of Al (2.5%) on the GR sample is related to the sandblasting procedure.

Table 2 includes the topography parameters of the surfaces. Focusing on the height parameters, the root mean square height of the surfaces (Sq) covers from low values for Mach and TiN, in the range of ca. 0.10 to 0.15 *μ*m up to the much rougher SB+AE sample, whose Sq attains ca. 3.4 *μ*m. The surfaces have almost zero skewness (Ssk), meaning that the heights of peaks and valleys are distributed symmetrically. AEt surface skewness though, is slightly negative, implying more valleys or cavities than peaks. According to the kurtosis (Sku) parameter, height distributions are Gaussian only for the SB+AE sample (Sku = 3.4 ± 0.4), but narrower than the Gaussian for the other samples. Kurtosis parameter for TiN and AEt (Sku ≈ 15) indicates that these surfaces have sharper peaks or deeper valleys than the others. Kurtosis input is in agreement with the relationship between the maximum height of the peaks (Sp), the valleys (Sv), the maximum height of the surface (Sz = Sp + Sv) and the arithmetical mean height of the surface (Sa). In SB+AE surfaces (Sku ≈ 3), the highest peak or valley is five times the Sa value, ten times in Mach and AEn surfaces (Sku ≈ 5), and 20 times in TiN and AEt surfaces (Sku ≈ 16). It can be noted that Mach and AEn surfaces are similar regarding SSk and SKu, but the height of features at AEn surfaces is seven times that of the Mach surface.

Regarding the spatial parameters, the auto-correlation length (Sal) is a measure of the distance to the next location of the surface with minimal correlation (s = 0.2 by default) with the original site. Is a quantitative measure as to the distance along the surface by which one would find a texture that is statistically different from the original location. Sal is about 1.5 *μ*m for Mach, TiN, and AEn samples, but it is two times higher for AEt and attains 7 *μ*m for SB+AE surfaces. These values indicate that SB+AE or AEt surfaces display a more significant separation among peaks than the other samples. However, we find a more isotripic distribution of features in these two surfaces (Str texture ratio close to 1) than in the other surfaces.

The hybrid parameter, the developed interfacial area ratio (Sdr), is a relevant roughness parameter for cell adhesion because it estimates the projected area from the measured one. In our samples, projections range from 1% to 200% of the measured area.

### 3.2. Cell Studies

**Figure 1:**
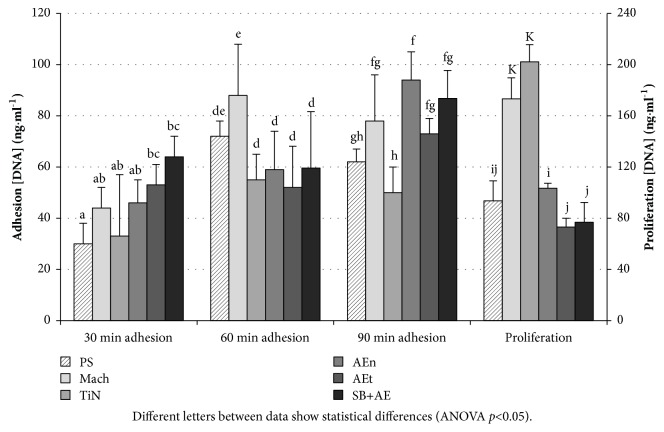
Primary fibroblast-like cells cultured on the test surfaces. Adhesion was measured at 30, 60, and 90 minutes of contact of the cells with the surfaces. Proliferation was measured at 72 hours of culture. Statistical analysis has been performed for each time of cell adhesion and proliferation independently. Different letters between data show statistical differences (ANOVA p<0.05).

Figure 1 shows the results of fibroblast adhesion and proliferation. Adhesion tends to reach higher values on the titanium substrates than on the PS control. Besides the PS control, the surfaces whit lower Sq values seem to promote significantly more cell attachment, especially at 60 min, when Mach yield substantially higher adhesion than AEt and SB+AE. Despite the larger Sal values at AEt and SB+AE, the peak heights may represent at these surfaces a fakir bed of nails for the cells, whose size is around one order of magnitude larger than distance of the topographical motifs, reducing the overall area and force of adhesion between the cell's cytoplasm and the rough surfaces. This is in agreement with other studies, which also found better adhesion and proliferation on plain surfaces than on rough surfaces [6]. At 90 min of adhesion, the amount of fibroblasts tends to equalize for all surfaces. This is in accordance with the creation of a cell microenvironment at the surfaces in which no further adhesion differences can be ascertained [36].

Cell proliferation at 72h of culture follows the trend already observed in adhesion experiment: smooth surfaces allow a significant higher level of fibroblasts proliferation than the rougher ones following TiN > Mach > AEn > SB+AE and AEt. The higher number of early adherent functional cells on smoother surfaces is not enough to explain these differences in proliferation. Rather, the high aspect ratio of the topographical cues may limit the process of proliferation at rougher surfaces with respect to the Mach and TiN surfaces.

**Figure 2:**
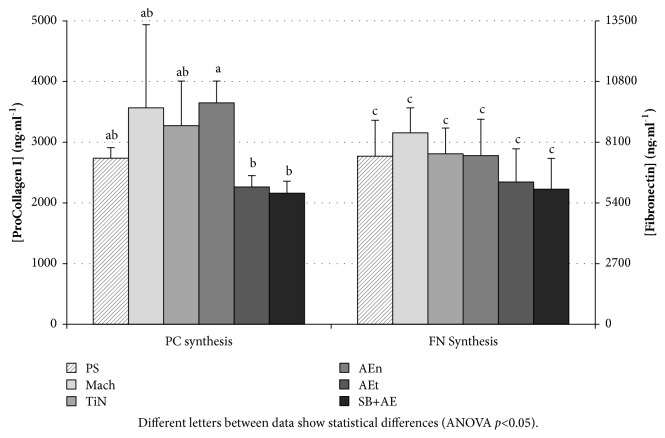
Synthesis of the fibroblast markers procollagen I (PC) and fibronectin (FN) on the test surfaces. Synthesis was measured at 7 days of culture. Statistical analysis has been performed for PC and FN synthesis independently. Different letters between data show statistical differences (ANOVA p<0.05).

Figure 2 shows the results of the fibroblasts autocrine synthesis of fibronectin (FN) and procollagen I (PC), which indicate that the ability of the cells to construct the extracellular matrix goes along with the aforementioned results. There is significantly more synthesis of PC at smooth surfaces than at rough surfaces. However, in this case, AEn surfaces, which may be considered as a smooth surface amongst the rough ones, reach values similar to that of the smoother Mach and TiN surfaces. While the roughness studied produces a less welcoming substrate for fibroblasts cell attachment and proliferation, AEn may demonstrate that certain topographical cues are capable of stimulating cell autocrine synthesis even if they are not as effective as the smoothest surfaces to make cells adhere and proliferate.

### 3.3. Microbial Studies

**Figure 3:**
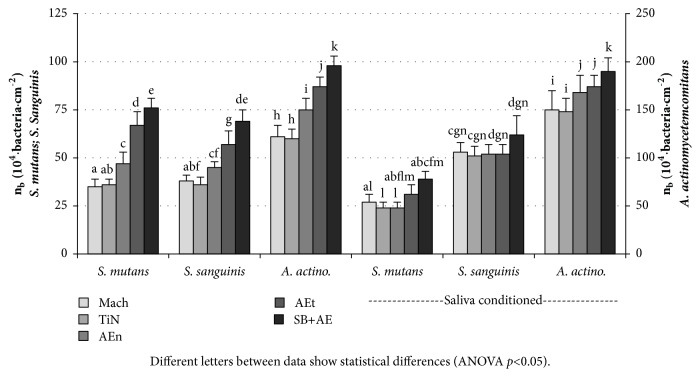
Bacterial adhesion per unit of area of* S. mutans*,* S. sanguinis*, and* A. actinomycetemcomitans* measured at 60 min of contact on the test surfaces. One set of samples was in contact with artificial saliva only and the other, with natural saliva. Different letters between data show statistical differences (ANOVA* p*<0.05).

Figure 3 shows the number of bacteria per unit of area of the samples studied. In general, bacterial adhesion increases following Mach ≈ TiN < AEn < AEt < SB+AE. Adhesion of* A. actinomycetemcomitans *is always higher than* S. mutans *or* S. sanguinis* in any given condition. In the absence of natural saliva conditioning, the adhesion of* S. mutans *and* S. sanguinis* is similar, except for the AEt surface, with a lower retention of* S. sanguinis* than* S. mutans*. For the three strains, adhesion on Mach and TiN coincides and increases significantly on AEn, AEt, and SB+AE. Only in the case of* S. sanguinis* on AEn the adhesion is similar to the adhesion on Mach or TiN. Under natural saliva conditioning,* S. mutans* adhesion on every surface finish, except on Mach, approximately decreases down to the adhesion measured on Mach surface. The natural saliva conditioning renders the adhesion of* S. sanguinis* on all the samples identical, at the same level that on the AEt surface without natural saliva conditioning. In the case of* A. actinomycetemcomitans* adhesion increases on the smoother surfaces, Mach, TiN, and AEn, but remains unchanged on the rougher samples, AEt and SB+AE.

**Figure 4:**
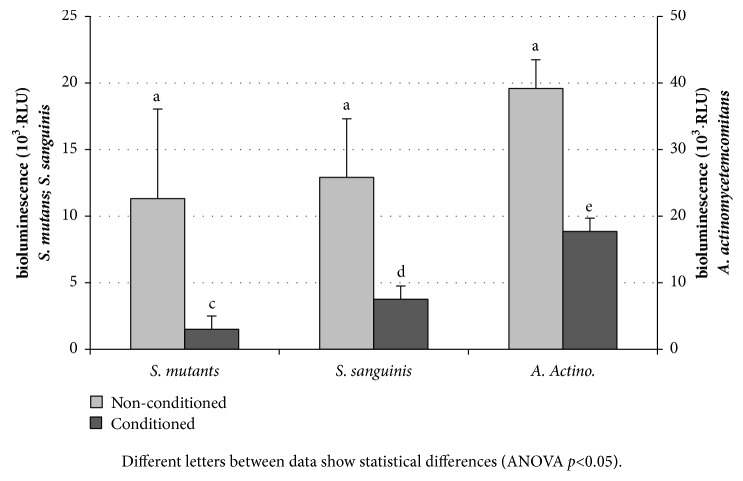
Viability biofilm formation of* S. mutans*,* S. sanguinis* and* A. actinomycetemcomitans* ATCC on nonconditioned samples and conditioned with human natural saliva. Results of growth after 24 hours, measured in relative light units (RLU). Different letters between data show statistical differences (ANOVA p<0.05).

The viability of the bacterial biofilms at 24 hours varied with the strain studied:* S. mutans* and* S. sanguinis* produced less bioluminiscence than* A. actinomycetemcomitans. *No statistically significant differences were found between the URL averages of the different roughness studied and this also when conditioned with natural human saliva. The composition of the saliva, however, affected considerably the biofilm viability, being much lower in the saliva-conditioned samples, irrespective of the surface conditions. The average value of biofilm viability on all surfaces after 24h of culture on the surfaces is shown in Figure 4.

SEM micrographs showed differences in biomass distribution for each surface and bacterial species. However, these results provide no information about the viability of the bacteria included in the biofilm. Images of the biofilms formed on Mach and on SB+AE surfaces, as representatives of the most dissimilar topographies examined, are shown in Figure 7. In all cases, the biomass found at the saliva-conditioned samples was significantly lower than at the nonconditioned. Biomass was also clearly higher on the rough surfaces than on the smooth ones, irrespective of the conditioning.

## 4. Discussion

The particular physiological processes of the different tissue compartments in contact with the implant/prosthetic component have stirred the design of surfaces with different textures.

In this context, the transepithelial component surface and the implant neck or body surfaces can be regarded as major subsystems in which it makes sense to customize the surface characteristics to the particular surrounding tissues. These components may interface the soft tissue of the mucosa, in which the major cellular component is the fibroblast. Therefore, there is need of a balance of surface properties that, on the one side stimulates cell adhesion and, on the other, inhibits bacterial presence. This is so because these parts are in contact with soft or hard tissue but also with the oral cavity when there is marginal gingival or bony tissue loss. Taking into account that the oral cavity is one of the most complex and populated bacterial system in the human body and that the osseointegrated portion of the neck concentrates most of the biomechanical interest of the implant, the challenge is set.

In this work we analyse the reaction of primary human gingival fibroblasts and three representative bacterial species of infectious processes in the oral/implant environment on differently treated surfaces for implants and implant components. Their texture has been characterized by optical profilometry, according to ISO 25148. This characterization provides a complete topographical analysis to better understand the behaviour of cells and bacteria in the surfaces tested, since it is known that these cells display dissimilar adhesion strategies [37].

The root mean square gradient of the surface, Sdq, provides information on how steep the surface slopes are. It is affected by the height and the fastest decay autocorrelation rate or distance among features in surface. For instance, a large Sdq would reflect a landscape of nearby sheer features. Nevertheless, within the experimental uncertainty, none of the topographical differences among the surfaces appears to affect fibroblast adhesion (Figure 1). Not even the larger developed area ratio for the rougher samples, Sdr, favours adhesion. It appears that the increase in surface could not be sensed by fibroblast, probably because it takes place in a scale domain not accessible to cells.

Although increasing surface roughness does not affect greatly the number of adhered cells, it does reduce fibroblasts proliferation (Figure 1). The increase in Sdq, from 0.15±0.01 of the Mach surface up to 1.23±0.09 for the AEn sample, is associated with a decrease in fibroblast proliferation, close to 40%, and it gets up to 50% for AEt and SB+AE samples, whose Sdq ≈ 2. It seems that cell proliferation needs larger and, more importantly, flatter accessible areas than cell adhesion. The steep features of the rougher surfaces studied may hamper cell proliferation, while the better behaviour found on AEn samples may be related to the similar vertical topographical features (Ssk and Sku) found in Mach surfaces. Lacking substantial topographical differences with respect to Mach surfaces, the particular increase in proliferation at TiN surfaces may rather be due to its titanium nitride layer.

As for cell adhesion, topography does not seem to be a determining parameter for fibroblast differentiation, at least after 7 days of culture. Only the AEn surfaces reach a statistically significant increase in PC I synthesis with respect to rougher surfaces (Figure 2).

In contrast with the large-sized fibroblasts, bacterial adhesion should not be influenced by the relative height of the surfaces. The absolute bacterial adhesion to surfaces shown in Figure 3 is a good indicator of how the surface of a same transmucosal piece can determine more or less bacterial adhesion. In this regard, the rougher the surface, the more bacteria will be found on the piece due to the increase in the total available surface for adhesion.

In order to evaluate the balance between fibroblast and bacterial adhesion to the surfaces, we have used the developed area ratio, Sdr. This parameter provides information about the accessible surface area to bacteria to estimate the degree of surface occupation; that is, the areal density of adhered cells referred to the real surface of the samples available for occupation. This allows a systematic comparison of the adhesion to a given surface, irrespective of its topography. Being n_b_ the number of cells per unit of area (Figure 3), the number of adhered cells per unit of the real surface, n_b,N_, or the areal density of cells can be calculated as follows:

(1) $$\begin{array}{c} n_{b,N}=\frac{n_{b}}{\left[1+\left(Sdr/100\right)\right]} \end{array}$$

**Figure 5:**
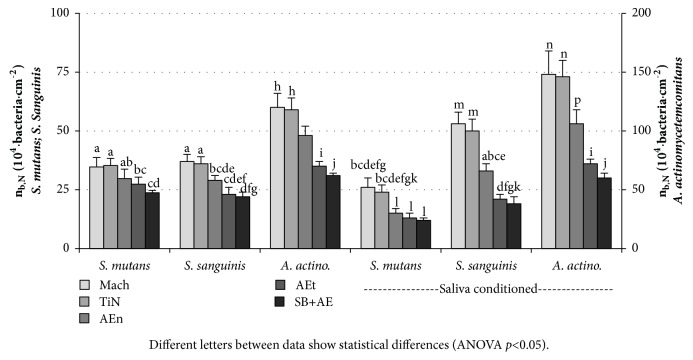
Areal density of bacteria (n_b,N_) of* S. mutans*,* S. sanguinis*, and* A. actinomycetemcomitans* on the test surfaces. One set of samples was in contact with artificial saliva only and the other, with natural saliva. Different letters between data show statistical differences (ANOVA* p*<0.05).

Figure 5 shows the areal density of bacteria (n_b,N_) for the three bacterial species considered, on surfaces conditioned or not with human saliva. In contrast with the behaviour observed for n_b_ (Figure 3), n_b,N_ is higher on the surfaces with lower Sq, Mach, and TiN, than on the rougher surfaces, when their Sdr reaches up to 200%. AEn shows an intermediate behaviour between the rougher and the smoother surfaces. This suggests that some parts of the measured area are not even available for bacteria to adhere. For the three bacterial species, the areal density of bacteria can be grouped in two distinct groups: the smooth Mach and NiT and the rougher AEt and SB+AE. Within each group, the areal density of bacteria depends on the bacterial strain and the conditioning with saliva or not, rather than on the kind of surface. Conditioning the surfaces with human saliva decreases the areal density of* S. mutans* on all the surfaces and while it does not affect the areal density of the other strains on the rough surfaces, it increases on the smooth surfaces.

Human saliva contains more than 2200 different proteins such as mucins, amylases, defensins, histatins, proline-rich proteins, statherins, lactoperoxidases, lysozymes, lactoferrins, and immunoglobulins, with different roles and effects within the salivary pellicle [38] and, thus, in bacterial adhesion. Among them, statherin has an inhibitory effect on* S. mutans* adhesion [39]. However, the reason behind the different behaviour observed is still unclear. Since the bacterial adhesion experiments were done under flow, we can speculate that bacteria at smoother surfaces may be more easily swept away than those on rougher surfaces, where the cavities may shelter the bacteria from the flow.

In contrast with the bacterial adhesion experiments, biofilm viability is not influenced by the different roughness of the surfaces. Almaguer Flores et al. reported a similar behaviour for* S. mutans* adhesion experiments. While for single bacteria adhesion events the authors reported dependence with surface roughness and hydrophobicity, they did not observe such reliance for biofilms [40]. With saliva conditioning we also found no roughness-related differences in biofilm viability, but an overall sharp reduction (Figure 4). The results obtained are in accordance with some studies on the viability and biomass present for mature biofilm existing in the presence of saliva [40–43]. Paradoxically, on resin composite the* S. mutans* biofilm formation in the presence of saliva was greater on surfaces that had deeper and larger depressions than with smaller and less deep roughness [44]. The organization of cells in the biofilm is a relevant characteristic as it can have an impact in the biofilms susceptibility to antimicrobial agents [45]. Figure 7 shows that the biofilm architecture was affected by the saliva conditioning. Salivary components have great impact not only in bacterial adhesion, but also in the ability to form biofilms efficiently [46]. The presence in saliva of some antimicrobial substances such as lysozyme, lactoferrin, lactoperoxidase, and secretory IgA [47] can be related to the modifications observed in the biofilms viability and biomass.

The A.* actinomycetemcomitans* biofilm formed on the surfaces produces higher values of bioluminescence than the streptococcus strains either conditioned or not with saliva (Figure 4), in accordance with Almaguer-Flores et al. 2012 [40]. The greater amount of biofilm might be due to a high biofilm compaction and production of amorphous extracellular material on surfaces as observed in the micrographs (Figure 7). In the SEM images apparently the biofilm of* S. mutans* seems larger than the* S. sanguinis* strains (Figure 7). The interactions between salivary agglutinin and the P1 adhesin of* S. mutans* contribute to bacterial aggregation and mediate the adhesion sucrose-independent to tooth surfaces [46].

The micrographs allow us to observe the biomass generated on the samples surfaces, but many bacteria inside of biofilm may be dead or dormant. This situation has already been cited in other articles [48]. They observed a high percentage of dead cells among the adhering bacteria on all titanium samples after 12 h of incubation. The authors argue the pivotal role of dead biological material in the formation process of (oral) biofilms. Similar results were observed in another study, where about 40% of bacteria were dead after different intraoral incubation times [49]. We have verified the difference between the biomass formed and the bacterial viability after different incubation times (Results not shown). It was confirmed the reduction of biofilm bacterial viability on surface, when the cultures were maintained for 48 to 72 hours. However, the amount of biofilm formed on the surface was increasing. Similar results were obtained in other studies [50], where the amounts of adherent biofilm increased during the incubation in a time-dependent manner.

**Figure 6:**
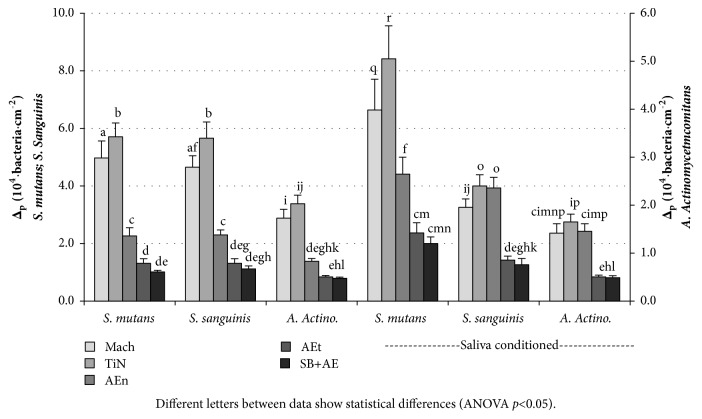
Ratio of fibroblasts proliferation, after 60 minutes of contact, to the number of bacteria of* S. mutans*,* S. sanguinis*, and* A. actinomycetemcomitans* in the projected area on the test surfaces. One set of samples was in contact with artificial saliva only and the other, with natural saliva. Different letters between data show statistical differences (ANOVA p<0.05).

**Figure 7:**
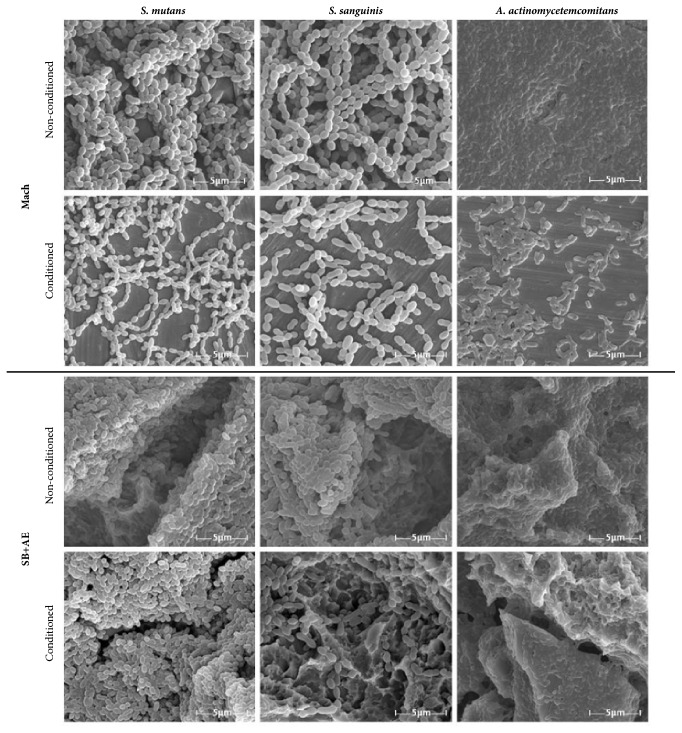
SEM micrographs of the biofilm formation of* S. mutans*,* S. sanguinis*, and* A. actinomycetemcomitans* on Mach and SB+AE surfaces. All images shown were taken at magnification 15000x and were chosen as the best representatives of the amount of biofilm on surfaces.

Although the experiments with fibroblasts and bacteria were carried out separately, we have condensed both data and compared how surfaces perform for cells and bacteria simultaneously.  Figure 6 shows the ratio of fibroblasts proliferation, ∆_p_, after 60 minutes of contact to the number of bacteria in the projected area. Since cell adhesion is not sensitive to surface roughness, the ratio adhered cells/bacteria will have the same behaviour as that the indicated by bacterial adhesion. However, the ratio of fibroblast proliferation to bacterial adhesion, ∆_p_, (Figure 6) is highly positive for TiN surfaces in particular, followed by Mach and AEn surfaces in this order. Among the rough surfaces, AEn displays the better behaviour. AEt and SB+AE surfaces do not show significant different behaviours. These ratios are irrespective of the saliva conditioning.

The more cells adhere and proliferate on the surface, the better the implant integration becomes. On the other hand, just a small number of bacteria can be enough to overcome the immunological defences and originate an infection. The winner of* the race for the surface* will determine the fate of the implant [51]. Although the rougher surfaces seem in the losing end in this race, they also display different levels of available surface for the two families of cells studied. Further experiments combining mammalian cells and bacteria would be needed to shed more light on this matter.

## 5. Conclusions

Implant or prosthetic component surfaces with different topographies in the micron and submicron level were subjected to gingival fibroblast cell adhesion and proliferation as well as bacterial adhesion and biofilm formation of three representative strains involved in peri-implant oral diseases.

While gingival fibroblast adhesion was similar in all surfaces increasing surface roughness reduced fibroblasts proliferation and increased the absolute bacterial adhesion and biofilm biomass. However, the results of adhered bacteria per unit area of developed surface suggest that some parts of the measured areas are not available for bacterial adhesion.

Saliva-conditioned samples showed significant less biofilm viability and biomass than the nonconditioned samples, which indicates that biofilm maturation is highly mitigated by the presence of salivary proteins.

To sum up, the ratio of fibroblast proliferation to bacterial adhesion is highly positive for TiN surfaces and Mach and AEn surfaces in this order. Accordingly, these surfaces may produce a better balance between tissue integration and infections of bacterial origin, helping in this way to improve the results of the clinical practice.

## Figures and Tables

**Table 1:** XPS Elemental Composition (at%) of samples: Mach, TiN, AEn, AEt, and SB+AE.

	Mach	TiN	AEn	AEt	SB+AE
Ti2p	18.34	23.44	17.50	6.80	16.62
O1s	50.07	25.67	49.57	48.31	49.32
C1s	29.14	22.26	30.98	27.98	28.93
N1s	1.48	27.98	1.24	2.29	1.25
P2p	0.32	0.39	0.19	14.20	0.50
Al2p					2.51
Si2p	0.27		0.41		0.31
Ca2p	0.14			0.43	0.15
Cl2p	0.14	0.10			
Ba3d	0.09	0.02			
F1s					0.41
S2p		0.14	0.10		

**Table 2:** Topography parameters of surfaces: Mach, TiN, AEn, AEt, and SB+AE.

	Mach	TiN	AEn	AEt	SB+AE
Sq (*μ*m)	0.099 ± 0.005	0.154 ± 0.005	0.63 ± 0.06	1.2 ± 0.1	3.4 ± 0.4
Ssk	-0.2 ± 0.4	0 ± 1	-0.1 ± 0.2	-0.6 ± 0.4	0.1 ± 0.1
Sku	5 ± 2	15 ± 4	5 ± 1	17 ± 3	3.4 ± 0.4
Sp (*μ*m)	0.6 ± 0.1	1.9 ± 0.2	4.6 ± 0.1	13 ± 5	14 ± 2
Sv (*μ*m)	0.8 ± 0.3	2.12 ± 0.07	5.5 ± 0.7	16.0 ± 0.8	14 ± 4
Sz (*μ*m)	1.5 ± 0.4	4.0 ± 0.1	10.1 ± 0.8	29 ± 4	28 ± 6
Sa (*μ*m)	0.077 ± 0.002	0.108 ± 0.002	0.48 ± 0.04	0.82 ± 0.04	2.7 ± 0.3
Sal (*μ*m)^*∗*^	1.31 ± 0.03	1.74 ± 0.06	1.50 ± 0.05	3.1 ± 0.3	7.5 ± 0.3
Str^*∗*^	0.035 ± 0.007	0.14 ± 0.06	0.61 ± 0.03	0.884 ± 0.001	0.79 ± 0.06
Sdr (%)	1.0 ± 0.1	2.2 ± 0.4	58 ± 7	145 ± 18	220 ± 110
Sdq	0.15 ± 0.01	0.22 ± 0.02	1.23 ± 0.09	2.0 ± 0.1	2.5 ± 0.7

*∗*Sal and Str parameters with s = 0.2 by default.

## Data Availability

The data used to support the findings of this study are included within the article.
